# Floating Mat Formation Makes *Zizania latifolia* More Competitive under the Conditions of Continuous Significant Water Level Rise

**DOI:** 10.3390/plants12051193

**Published:** 2023-03-06

**Authors:** Ji-Hui Wen, Bing-Yao Li, Hong-Yu Xiao, Cai-Ying Gong, An-Guo Gao, Yan-Hong Wang, De-Liang Li, Hong-Yuan Zeng, You-Zhi Li, Gui-Xiang Yuan, Hui Fu, Ai-Ping Wu

**Affiliations:** 1 Ecology Department, College of Resources and Environment, Hunan Provincial Key Laboratory of Rural Ecosystem Health in Dongting Lake Area, Hunan Agricultural University, Changsha 410128, China; 2 State Key Laboratory of Subtropical Silviculture, Zhejiang A&F University, Hangzhou 311300, China; 3 Hunan Engineering Technology Research Center of Featured Aquatic Resources Utilization, College of Animal Science and Technology, Hunan Agricultural University, Changsha 410128, China; 4 Hunan Institute of Microbiology, Changsha 410009, China

**Keywords:** aquatic plant, floating mat, emergent macrophyte, uprooting, angle, environmental filter, deep water, survival strategy

## Abstract

Water level rise is considered an environmental filter for the growth and reproduction of aquatic plants in lakes. Some emergent macrophytes can form floating mats, enabling them to escape from the negative effects of deep water. However, an understanding of which species can be uprooted and form floating mats easily and what factors affect these tendencies remains greatly elusive. We conducted an experiment to determine whether the monodominance of *Zizania latifolia* in the emergent vegetation community in Lake Erhai was related to its floating mat formation ability and to try to find the reasons for its floating mat formation ability during the continuous increase in water level over the past few decades. Our results showed that both the frequency and biomass proportion of *Z. latifolia* were greater among the plants on the floating mats. Furthermore, *Z. latifolia* was more likely to be uprooted than the other three previously dominant emergent species due to its smaller angle between the plant and the horizontal plane, rather than the root:shoot or volume:mass ratios. The dominance of *Z. latifolia* in the emergent community in Lake Erhai is due to its easier ability to become uprooted, allowing it to outperform other emergent species and become the single dominant emergent species under the environmental filter of deep water. The ability to uproot and form floating mats may be a competitive survival strategy for emergent species under the conditions of continuous significant water level rise.

## 1. Introduction

Subtropical shallow lakes are subject to water-level fluctuations due to both natural climate variability and anthropogenic regulation [1]. The structure and function of these lake ecosystems are greatly affected by these fluctuations because macrophytes, the main primary producers in the lakes, are very sensitive to water level change [2]. Accordingly, the health and integrity of the lake ecosystem can be strongly impacted by any significant water level change [2,3]. In lakes, water level rise is a very common stressor on the growth and reproduction of aquatic plants, and the stress is characterized by its rate, duration, frequency, amplitude and timing [3,4,5]. Macrophytes usually develop physiological and morphological adaptations to flooding [2,6,7]. In general, aquatic plants adopt three survival strategies in response to flooding depending on the water depth. First, plants can develop faster elongation to escape from the water when coping with partial submergence [8]. The second strategy, called quiescence, is formed when long periods of complete submergence happen [6]. Third, some plants can tradeoff between an escape strategy and a quiescence strategy based on the depth of the flood water [9]; however, all three of these survival strategies are energy-consuming [10]. If the flooding continues or increases, flooded plants will ultimately exhaust their reserves, and then they will be severely damaged and even die [10].

Based on the above-mentioned findings, we can conclude that the three survival strategies cannot function effectively under conditions of deep submergence or a long duration of flooding, especially for emergent plants. These species are confined to a narrow strip in the littoral area mainly because of their low flood tolerance, though the competition from neighbors also plays a small role [11]. However, floating mats enable emergent macrophytes to escape from the negative effects of fluctuating water levels, especially in deep water [11,12,13,14]. The formation of floating mats is a double-edged sword for emergent plants. On one hand, the potential damage caused by water currents and wind waves is increased; on the other hand, deep flooding is avoided [11]. However, due to their many advantages over traditionally constructed wetlands, many artificial floating treatment wetlands have been constructed to treat domestic, industrial and agricultural wastewater and eutrophic water [12,13,14]. Moreover, floating mats can also provide a safe habitat for vulnerable and endangered plant species [15,16]. Although emergent mat formation is a global geographical phenomenon [17], only some common emergent macrophytes, such as *Phragmites*, *Zizania*, *Typha*, *Scirpus*, *Carex*, *Cladium*, *Cyperus* and *Vossia*, can develop floating mats [5,15,18]. Therefore, we can speculate that a species that can form floating mats more easily would have a superior competitive advantage over others and could dominate the emergent community under conditions of a substantial water level rise. Nevertheless, to our best knowledge, there has been no research on this topic. Moreover, the reason why these emergent plants form floating mats more easily than the other plants is not well documented.

In general, the formation of an emergent floating mat demands two habitat requirements: shelter from wind and wave action and stability of water level fluctuations [11]. The adaptations of macrophytes to deep water include increases in plant height, branch length, shoot internodes and shoot:root biomass ratio, and reductions in belowground biomass and branch number [2,6,7,19,20]. All these adaptations can be demonstrated by the root:shoot biomass ratio of a plant to some degree. These responses result in a reduction in root investment and a shallow root system for emergent species [21], which means that the root anchorage strength of these emergent plants is greatly weakened, although the root function might be more effective than ever before [22]. Thus, anchorage failure or breaking failure of the stems is inevitable due to the reduction in root anchorage strength and contributes to floating mat formation [23].

Aquatic species typically have extensive aerenchyma in their shoot, root and rhizome tissues, which enhances their buoyancy [13,14,15]. A macrophyte with more extensive aerenchyma in tissues will have a greater volume-to-mass ratio, and thus greater buoyancy, based on Archimedes’ principle. Accordingly, the greater buoyancy a macrophyte has, the higher the possibility of the plants being uprooted, and uprooted plants are important components of floating mats [11]. It is well known that wind and wave action greatly affect whether an aquatic plant becomes uprooted [23]. Usually, the force of winds and waves on the plant has two force components (a horizontal force component and a vertical force component) based on the angle between the plant and the horizontal plane [24]; however, only the vertical force component has the ability to uproot a plant, and the horizontal force component normally breaks the plant. Accordingly, the smaller the angle between the plant and the horizontal plane is, the greater the vertical force component is based on Newton’s laws of motion, and the plant is more likely to be uprooted.

**Figure 1:**
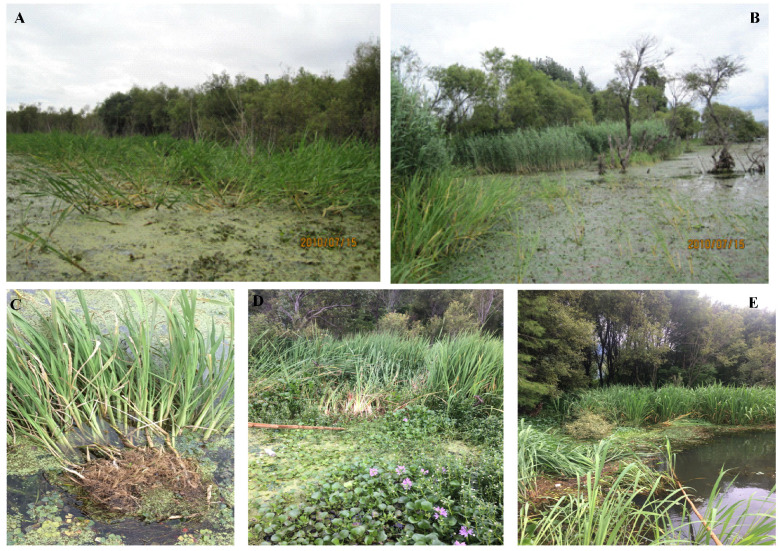
Natural patterns of *Z. latifolia* and *P.australis* (**A**,**B**, photos by Bi-Bi Ye) and scenes of floating mats (**C**–**E**, photos by Ya-Xuan Zhao) in Lake Erhai.

As far as plants are concerned, we believe that the uprooting or the floating mat formation of an emergent macrophyte is greatly influenced by the three factors (the plant root:shoot biomass ratio, the plant volume:mass ratio, and the angle between the plant and the horizontal plane) mentioned above, though other mechanisms may play important roles in these functions [23,24,25]. In our field survey, we found that the emergent community in Lake Erhai had changed from a *Phragmites australis*, *Typha orientalis* and *Acorus calamus* polydominant community to a *Zizania latifolia* monodominant community over the past decades during a long period of increasing water level, usually over 80 cm (and even 100 cm in some specific years) than before since 2003 by water level regulation, [20,26,27]. Furthermore, an increasing number of floating mats had formed and distributed throughout the lake’s littoral zone (Figure 1). We want to determine whether the dominance of *Z. latifolia* is related to a higher tendency to uproot and form floating mats than is seen in other emergent plants. To address this knowledge gap, we conducted a study to determine which emergent species was dominant in the floating mat community, and the reason for this emergent species being more easily uprooted to form floating mats, as emergent macrophytes are the most important components of floating mats [11,16]. We hypothesized that (1) *Z. latifolia* dominated the floating mat community, which accounted for its dominance in the emergent community as the emergent plants on the floating mats can attach to the soil at the landward [11]; (2) the dominance of *Z. latifolia* in the emergent community in Lake Erhai was due to its easier tendency to become uprooted because of its lower plant root:shoot biomass ratio, greater plant volume:mass ratio and smaller angle between the plant and the horizontal plane than those of the other three previously dominant emergent macrophytes (*P. australis*, *T. orientalis* and *A. calamus*).

## 2. Materials and Methods

### 2.1. Study Area

Lake Erhai (25°36′–25°58′ N, 100°06′–100°18′ E) is located in Yunnan Province, Southwest China. It is a plateau lake, 42.58 km long and 8.0 km wide, with a 251.0 km^2^ water surface area and a 2656 km^2^ watershed. The study area is located in a warm plateau climate with an average annual temperature of 15.7 °C, a maximum temperature of 34.0 ℃ and a minimum temperature of −2.3 °C. The annual precipitation, sunshine duration and frost-free period are 1024 mm, 2345 h and 228 days, respectively. This region is mainly influenced by the southeast monsoon; the southeast wind prevails during the daytime, and the southeast and southwest winds prevail at night [28].

### 2.2. Field Survey

We recorded all of the floating mats (except the floating mats mainly consisting of floating-leafed macrophytes) along the littoral zone of Lake Erhai in summer (1973.10 m above mean sea level) and winter (1974.09 m above mean sea level), in 2017. The number of species, species identity, frequency and biomass proportion of each plant on these floating mats were determined. In each floating mat, one to twelve 1.0 × 1.0 m^2^ plots (48 in summer and 65 in winter) were randomly established based on the area of the floating mat. All the species were identified, and the biomass of each species was weighed after rinsing off all sediment (soil) and oven-dried at 70 °C for 72 h. The whole weight of the floating mat and the weight of every species on the floating mat were calculated based on the sample plots. To analyze the dominance of species on the floating mats, the biomass proportion of each species was calculated as the total biomass of the individual species divided by the total biomass of all the surveyed floating mats. Similarly, the frequency of each species was calculated as the number of times the species occurred on the floating mats divided by the total number of floating mats.

In August 2018, the angles between the plant and the horizontal plane of *Z. latifolia*, *P. australis*, *T. orientalis* and *A. calamus* at depths of 30, 60 and 90 cm were determined. For each species at each depth, six plants were randomly selected to measure the angles between the plant and the horizontal plane; the plants were then collected to determine their root:shoot biomass ratio and plant volume:mass ratio. Every plant was separated into roots and shoots, and their wet weights were determined. The shoot was put directly into a container full of water; the overflowing water was collected and measured with a graduated cylinder. The volume of the overflowing water was considered the volume of the shoot, based on Archimedes’ principle. The volume:mass ratio of the plant was calculated as the volume of the shoot divided by the shoot’s wet weight. After that, the root and shoot were dried at 105 °C for 30 min, oven-dried at 70 °C for 72 h, and weighed. The root:shoot biomass ratio of each plant was calculated as the root dry weight divided by the shoot dry weight.

### 2.3. Pot Experiment

In June 2018, seedlings (approximately 70 cm in length and similar in morphology) of the four study species were bought from a nursery of a local aquatic company. In every pot, four individuals of the same plant species were planted 10 cm deep for 4 weeks to establish root anchorage. Every pot had a circular area of 1962.5 cm^2^ (soil surface), 30 cm depth and pre-weighted 36 kg of sediment from the west littoral zone of Lake Erhai (organic matter: 11.6–15.3 g kg^−1^; total nitrogen: 1.34–1.65 g kg^−1^, total phosphorus: 0.21–0.36 g kg^−1^ and pH: 6.34–6.86, approximately 25 cm thick soil layer). These pots were watered to a 5 cm water depth and put on the ground with full light during the anchorage period. At that time, the heights of *Z. latifolia*, *P. australis* and *T. orientalis* were approximately 100–115 cm and the height of *A.calamus* was approximately 90–100 cm four weeks later.

The pots were randomly put in a small bay (25°43′5.07″ N, 100°11′38.41″ E) sheltered by a 150 m headwater channel on the west side of Lake Erhai, at depths of 40, 80 and 100 cm, respectively. Due to the limited height of plants, we used 100 cm rather than 120 cm as the third water level gradient. Each plant species at each depth was replicated six times. In this experiment, we used four species, three water depths, and six replicates, resulting in a total of 72 pots (288 plants). The uprooted plants were checked every day, and the experiment was terminated eight weeks later. The mean velocity of the wind was 4.3 m (a range of zero to eighteen m) every second, and the mean height of the wave was 0.5 m (a range of zero to 2.1 m) during the experiment time. The water level fluctuated between −5 cm and +25 cm during the whole study period (data from the Administration of Lake Erhai).

### 2.4. Data Analysis

Statistical data analysis of the raw data or transformed data (Pearson’s correlation analyses if necessary) was conducted using the software package R 3.5.2 [29]. Differences in root:shoot ratio, volume:mass ratio, and the angle between the plant and the horizontal plane among the three water depths and among the four emergent plants were compared by a two-way analysis of variance (ANOVA), followed by a Tukey HSD test. Multiple comparisons of each measured index were performed with a Tukey post hoc test. Homogeneity of variances was tested by using Levene’s test, and differences between means were deemed significant if *p*  <  0.05. To identify the factors affecting the uprooting tendencies of the four study species, relationships between the mean values of the root:shoot ratio, volume:mass ratio, the measured angle and frequency in summer, biomass proportion in summer, and mean uprooted percentage of the four species were analyzed by Pearson’s correlation analyses after the data were log(x + 1) transformed.

## 3. Results

### 3.1. Floating Mats

A total of eighteen floating mats, ranging from 2 to 10,050 m^2^ in summer and 22 floating mats ranging from 1.2 to 4560 m^2^ in winter, were recorded. Additionally, twenty-five aquatic and one terrestrial species (fifteen families and nineteen genera) were recorded in this survey (Table 1). Both the frequency and the biomass proportion of *Z. latifolia* were the highest among all of the recorded species in the floating mats in the two seasons (frequency: 73.33% in summer and 66.67% in winter; biomass proportion: 43.38% in summer and 41.91% in winter) (Table 1). Furthermore, the other five species with the highest frequencies and biomass proportions were *E. crassipes*, *A. philoxeroides*, *C. demersum*, *P. maackianus* and *V. natans* rather than any other emergent macrophyte.

### 3.2. Factors Related to Plant Uprooting

**Figure 2:**
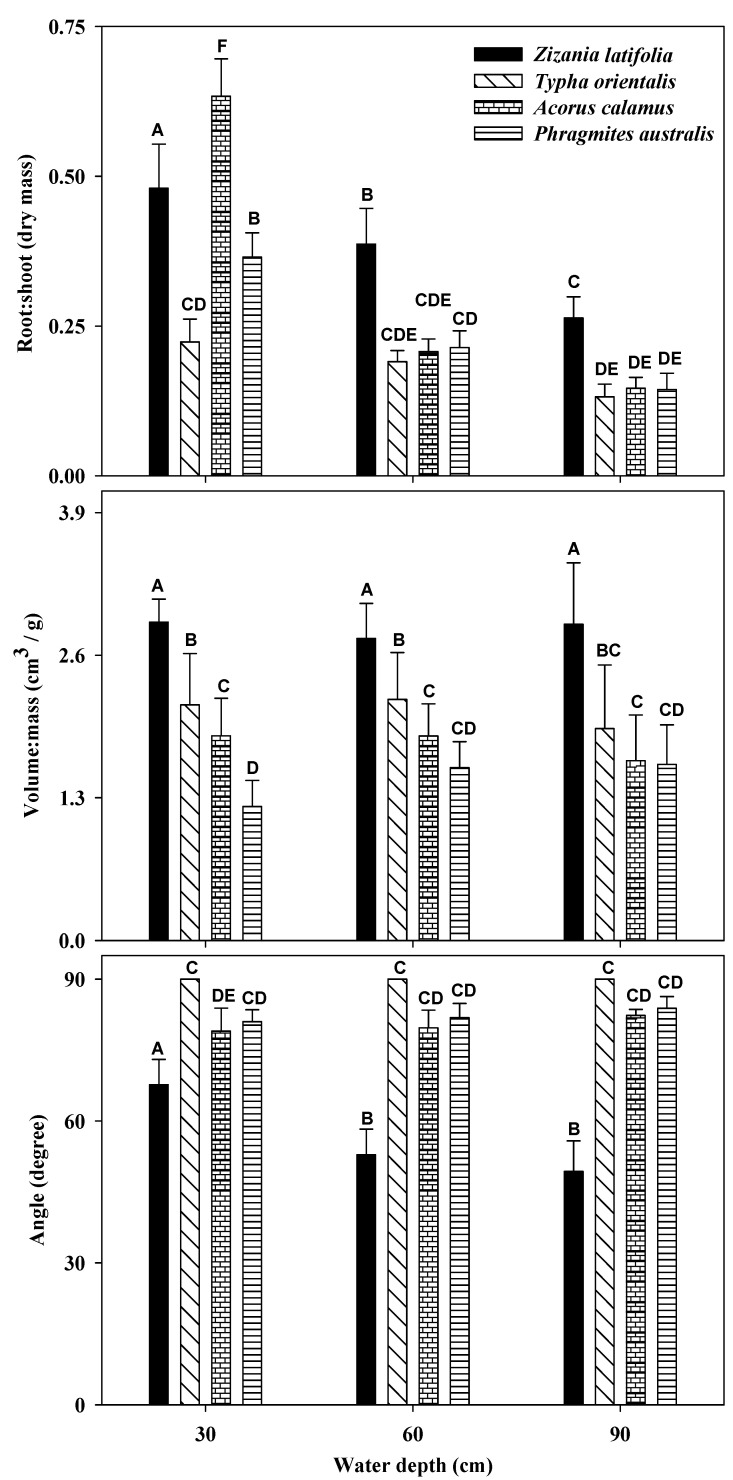
Root:shoot ratio, volume:mass ratio, and the angle between the plant and the horizontal plane of the four emergent macrophytes. Bars labeled with different letters mean significantly different.

Generally, the root:shoot ratio of all study plants decreased with water depth; however, the volume:mass ratio and the measured angle of the plants showed no significant differences among the different water depths, except the plant angle of *Z. latifolia* (Figure 2, Table 2). Regarding the three parameters, the root:shoot and volume:mass ratios of *Z. latifolia* were the highest among those of the four species at each water depth (except that the root:shoot ratio of *Z. latifolia* was lower than that of *A. calamus* at the water depth of 30 cm). In contrast, the measured angle of *Z. latifolia* was the smallest among those of the four species at each water depth (Figure 2, Table 2).

Given the relationships among the three measured characters and the floating mat indices of the macrophytes, the measured angle was significantly negatively correlated with the frequency and the biomass proportion but not with the percentage of plants uprooted (Table 3). However, neither the root:shoot ratio nor the volume:mass ratio was significantly correlated with the frequency, biomass proportion or percentage of plants uprooted, except that the volume:mass ratio was significantly correlated with the percentage of plants uprooted (Table 3).

### 3.3. Floating Experiment

**Figure 3:**
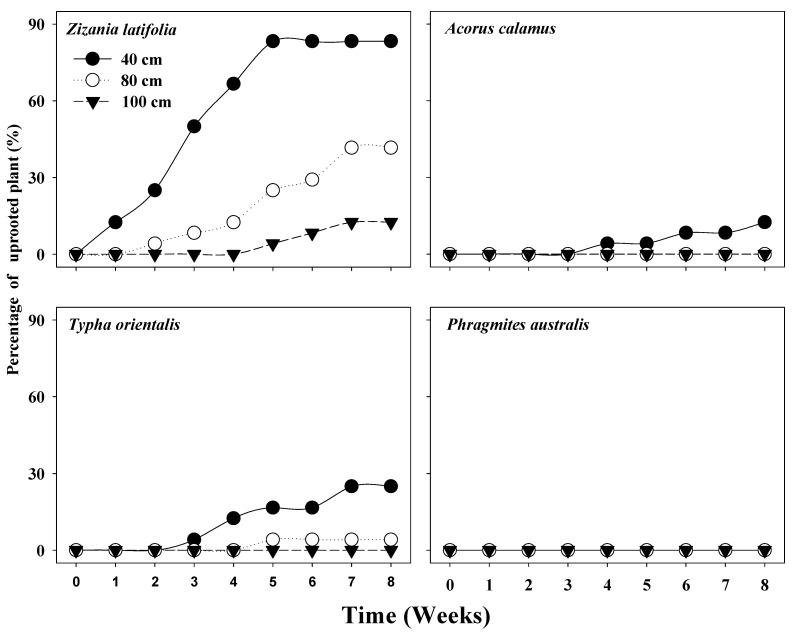
The percentage of plants uprooted of the four emergent macrophytes.

For all species, the highest percentage of plants were uprooted at the water depth of 40 cm, an intermediate percentage were uprooted at the water depth of 80 cm, and the lowest percentage of plants were uprooted at the water depth of 100 cm (Figure 3). Of the four surveyed species, the highest number of *Z. latifolia* plants was uprooted, followed by *T. orientalis* and *A. calamus* plants, and the lowest number of *P. australis* plants was uprooted. At the end of the experiment, the percentages of uprooted plants for *Z. latifolia* at water depths of 40, 80 and 100 cm were 83.33%, 41.67% and 12.5%, respectively; the percentages of uprooted plants for *T. orientalis* at water depths of 40, 80 and 100 cm were 25.00%, 4.17% and 0.00%, respectively; the percentages of uprooted plants for *A. calamus* at water depths of 40, 80 and 100 cm were 12.5%, 0.00% and 0.00%, respectively; no *P. australis* plants were uprooted (Figure 3).

## 4. Discussion

### 4.1. Dominance of Z. latifolia

Although the expansion of plants from the edge to the center of a lake can form floating mats, we observed that the floating mats in Lake Erhai were mostly formed from the uprooting of plants (Figure 1). Furthermore, our results indicated that *Z. latifolia* dominates the whole floating mats as observed (Figure 1), which implies that *Z. latifolia* is more easily uprooted than other aquatic plants. Our floating experiment results, likewise, clearly suggest that the ability of *Z. latifolia* to form floating mats is greater than that of the three formerly dominant emergent species (Figure 3).

The formation of floating mats enables *Z. latifolia* to escape from the stress of deep water in Lake Erhai [11,12,13,14]. Moreover, the emergent plants on the floating mats can attach to the soil at the landward’s end and grow as normal emergent macrophytes when the mats approach the lake shore by free-floating or by wind and wave action [11]. Accordingly, those emergent plants that can form floating mats more easily have many survivorship advantages over the other emergent plants. These advantages make *Z. latifolia* more competitive than the three formerly dominant emergent species in terms of deep-water stress and allow *Z. latifolia* to continue thriving in the lake-littoral zone. Then, *Z. latifolia* can outcompete the other emergent species, and ultimately become the dominant species, as observed in our study (Figure 1). For this reason, we can conclude that the dominance of *Z. latifolia* in the emergent communities in Lake Erhai is greatly owing to its strong tendency to form floating mats [26,27]. Additionally, the change from a *P. australis*, *T. orientalis* and *A. calamus* polydominant community to a *Z. latifolia* monodominant community over the past decades during a long period of significant water level rise is predictable and reasonable. Accordingly, we predict that the dominance of *Z. latifolia* in the emergent community and on the floating mats will be reinforced and more terrestrial plants (such as *Eupatorium adenophorum*) will grow on the floating mats if the water level rise continues in Lake Erhai. For the first time, we are reporting that the dominance of an emergent macrophyte is due to its strong tendency toward floating mat formation. Similarly, Zhang et al. reported that a *P. australis* and *Typha angustifolia* polydominant emergent community changed to a *Z. latifolia* monodominant emergent community in Lake Wuchang after the lake water level increased because of the construction of a sluice [30]. Furthermore, many previous experiments showed that *Z. latifolia* has greater flood tolerance than many other emergent macrophytes, such as *Scirpus tabernaemontani* and *T. orientalis*, and can outcompete them [31,32]. The dominance of *E. crassipes* and *A. philoxeroides* on the floating mats might be because they are aggressive invasive plants [33], while the dominance of *C. demersum*, *P. maackianus* and *V. natans* on the floating mats is in accordance with their dominance in submerged communities [34].

### 4.2. Factors Related to Plant Uprooting

Our results demonstrated that the root:shoot ratio of *Z. latifolia* was the greatest among the four tested emergent species, which suggests that *Z. latifolia* invests the most resources in its belowground growth [19,32]. However, the amount of energy used by *Z. latifolia* for its root anchorage deserves extensive research because a large portion of its belowground biomass is rhizomes, which account for its rapid expansion and asexual reproduction [30,35,36]. Furthermore, the rooting depth of *Z. latifolia* is the shallowest relative to its height among the four study species [19]. Accordingly, we do not think that the higher root:shoot ratio of *Z. latifolia* necessarily ensures that it has the strongest anchorage. Furthermore, the uprooting of an aquatic plant is caused by small gradual and continuous forces rather than a strong instant drag force, which may result in the plant breaking due to its limited mechanical resistance [26]. In other words, the uprooting of an aquatic plant is not primarily determined by its root anchorage.

In contrast, the high volume:mass ratio of *Z. latifolia* compared to those of the other tested species suggests that it has the most extensive aerenchyma in its tissues, which enhances its buoyancy and makes it float more easily [16,35]. In addition, *Z. latifolia* infected by *Ustilago esculenta* may have increased its buoyancy resulting from a greater volume:mass ratio [37]. The results for the volume:mass ratio of *Z. latifolia* agree well with much previous research [27,35,37,38]. The smallest angle between the plant and the horizontal plane of *Z. latifolia* indicates that the vertical force component of *Z. latifolia* is the greatest (Figure 1 and Figure 2), which means that *Z. latifolia* is more likely to be uprooted [24]. The plant is more likely to be broken if the horizontal component strength of the plant is much higher, as observed in the other three studied emergent species in our experiment.

Similarly, our results demonstrated that both the frequency and the biomass proportion of the studied plants were determined by the measured angle and that the percentage of plants uprooted was affected by the volume:mass ratio (Table 3). The results highlight that the uprooting and floating mat formation of emergent species are mainly determined by the angle between the plant and the horizontal plane. However, the percentage of plants uprooted was significantly correlated with the volume:mass ratio, not the measured angle, which implies that the plants in the pot experiment were uprooted primarily owing to their great buoyancy, rather than their angles (Table 3). These contrasting results might be because the duration of the pot experiment was very short and the plants were very young. Not enough tillers and rhizomes had developed in these young plants, which resulted in larger angles between the plant and the horizontal plane than those of wild mature plants (observed). Furthermore, the angle used for the correlation analysis was the measured angle obtained from wild mature plants rather than that obtained directly from the plants in the pots. In addition, the phenomenon that the percentage of plants uprooted was affected by the measured angle is very obvious, though the relationship between them was not significant enough (*R^2^* = 0.829, *p* = 0.089). Accordingly, we believe that the uprooting and floating mat formation of emergent species are mainly determined by the angle between the plant and the horizontal plane rather than by the root:shoot and volume:mass ratios of the macrophytes. It should be noted that the uprooting and floating mat formation of emergent species might be affected by its above-water level leaf area and biomass. However, an aquatic plant experiences a force in moving water more than 25 times greater than that in a wind of the same velocity [18]. Thus, we think that the effects caused by above-water level leaf area or biomass on the uprooting could be negligible compared with the forces exerted on the below-water level plant parts by waves and currents.

## 5. Conclusions

In conclusion, our results highlight that *Z. latifolia* tends to uproot and form floating mats more easily than the other three formerly dominant emergent macrophytes do. The reason is that the frequency and biomass proportion of *Z. latifolia* were the greatest among the plants on the floating mats. Moreover, the percentage of *Z. latifolia* plants uprooted was also the highest among the four emergent species. *Z. latifolia* becomes uprooted and forms floating mats easily because of the small angle between the plant and the horizontal plane, rather than because of its root:shoot or volume:mass ratios. Thus, we highlight that the dominance of *Z. latifolia* is related to its ability to uproot and form floating mats. The ability to uproot and form floating mats may be a new competitive survival strategy for emergent species under the conditions of continuous significant water level rise.

## Figures and Tables

**Table 1:** The frequency and biomass proportion of recorded species on floating mats in summer and winter. “\”: no data.

ID	Latin Name	Summer		Winter	
		Frequency (%)	Biomass Proportion (%)	Frequency (%)	Biomass Proportion (%)
1	*Alternanthera philoxeroide*	60.00	15.43	28.57	4.40
2	*Azolla imbricata*	13.33	\	9.52	\
3	*Ceratophyllaceae demersum*	53.33	0.61	47.62	0.18
4	*Ceratophyllaceae dubia*	20.00	2.32	0.00	0.00
5	*Ceratophyllaceae verticillata*	26.67	\	9.52	\
6	*Cynodon dactylon*	13.33	\	9.52	\
7	*Eichhornia crassipes*	60.00	9.99	47.62	31.16
8	*Eupatorium adenophorum*	6.67	\	0.00	\
9	*Leersia hexandra*	13.33	\	9.52	\
10	*Marsilea quadrifolia*	20.00	\	14.29	\
11	*Myriophyllum elatinoides*	13.33	0.83	19.05	11.91
12	*Myriophyllum spicatum*	26.67	\	19.05	\
13	*Nymphoides peltatum*	6.67	\	0.00	\
14	*Phragmites australis*	17.35	0.93	19.05	1.52
15	*Polygonum amphibium*	20.00	\	14.29	\
16	*Polygonum hydropiper*	13.33	\	4.769	3.79
17	*Potamogeton intortifolius*	0.00	\	4.769	\
18	*Potamogeton maackianus*	26.67	21.97	33.339	2.22
19	*Potamogeton malaianus*	26.67	\	9.529	\
20	*Potamogeton pectinatus*	6.67	\	0.00	\
21	*Spirodela polyrrhiza*	20.00	\	14.29	\
22	*Trapa incisa var. quadrispinosa*	26.67	2.87	19.05	0.00
23	*Trapa natans*	26.67	\	19.05	\
24	*Typha orientalis*	6.67	0.38	9.52	1.36
25	*Vallisneria natans*	26.67	17.24	33.33	4.45
26	*Zizania latifolia*	73.33	41.91	66.67	43.38

**Table 2:** ANOVA results of root:shoot ratio, volume:mass ratio, and the measured angle of the four emergent species at the three different water depths. Values of *p* < 0.05 are in bold.

Source	Root:Shoot			Volume:Mass			Angle		
	*df*	*F*	*P*	*df*	*F*	*P*	*df*	*F*	*P*
Species (S)	3	67.53	**0.000**	3	34.46	**0.000**	3	67.53	**0.000**
Water depth (WD)	2	199.86	**0.000**	2	0.25	0.781	2	1.48	0.236
S ×WD	6	28.96	**0.000**	6	0.90	0.501	6	3.12	**0.010**
Residual	60			60			60		

**Table 3:** Relationships among the measured angle, volume:mass ratio, root:shoot ratio and frequency, biomass proportion and percentage of plants uprooted of the four emergent macrophytes. Values of *p* < 0.05 are in bold.

	Frequency		Biomass Proportion		Percent Uprooted	
	*R* ^2^	*P*	*R* ^2^	*P*	*R* ^2^	*P*
Measured angle	0.916	**0.043**	0.946	**0.027**	0.829	0.089
Volume:mass	0.614	0.216	0.774	0.120	0.919	**0.042**
Root:shoot	0.419	0.353	0.533	0.270	0.861	0.072

## Data Availability

The data presented in this study are available on request from the corresponding author. The data are not publicly available due to privacy.
